# Supplementary figures and images for: T-cell responses to sequentially emerging viral escape mutants shape long-term HIV-1 population dynamics
Source: PLoS Pathog. 2020 Dec 28;16(12):e1009177. doi: 10.1371/journal.ppat.1009177 (PMC7833229; doi:10.1371/journal.ppat.1009177)

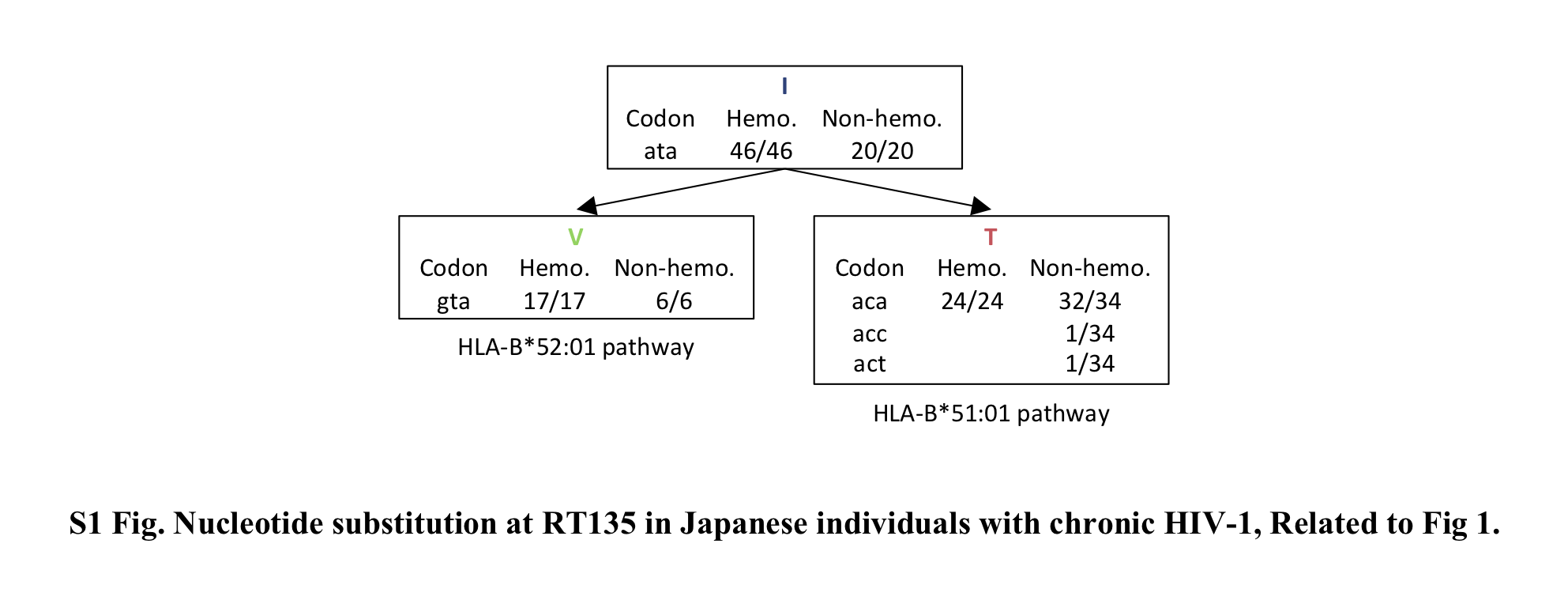

Supplement: S1 Fig — Codon usages for I, V, and T are shown, along with their frequencies observed in Japanese individuals with chronic HIV-1 (Hemo; 95 hemophiliacs shown in Fig 1A, Non-hemo; 83 non-hemophiliac individuals shown in Fig 1B and S1 Table). (TIF) [file ppat.1009177.s001.tif]

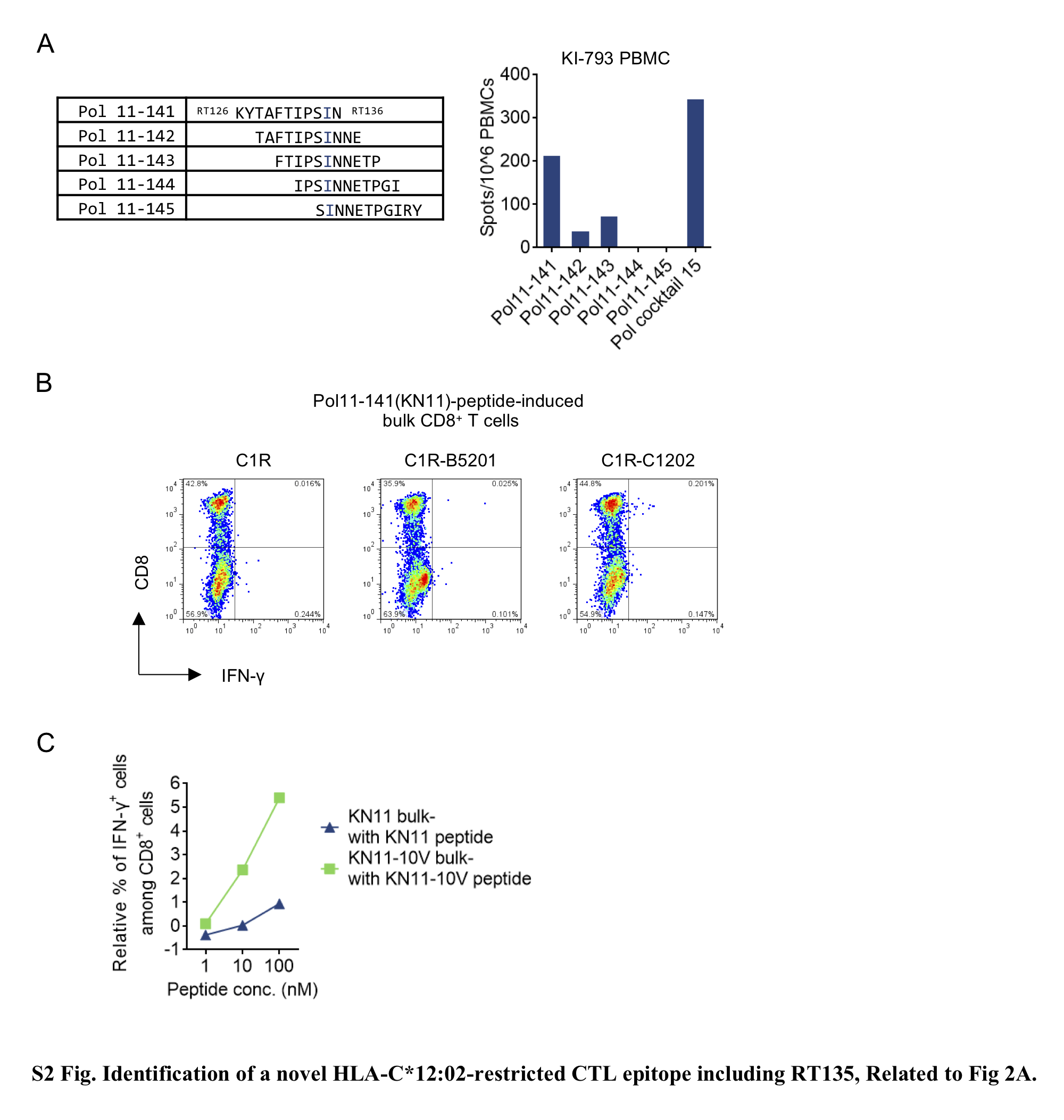

Supplement: S2 Fig — A. T-cell responses to 11-mer overlapping Pol peptides containing RT135. T-cell responses of PBMCs from an HLA-B*52:01+C*12:02+ individual (KI-793) to five 11-mer overlapping Pol peptides containing RT135 position and Pol peptide cocktail 15 including the 5 overlapping peptides were analyzed at a concentration of 100 nM by ELISPOT assay. B. Identification of HLA-restriction of the response to the Pol 11–141 (KN11) peptide. IFN-γ production from KN11-induced bulk T cells stimulated with C1R cells expressing either HLA-B*52:01 or -C*12:02 molecule pre-pulsed with the KN11 peptide at concentration of 100 nM was analyzed by ICS assay. C. Comparison of induction efficiency between bulk T cells induced with the KN11 peptide and those with KN11-10V mutant one. IFN-γ production from KN11-induced or KN11-10V-induced bulk T cells stimulated with C1R-C1202 cells pre-pulsed with the KN11 or KN11-10V peptide was analyzed by ICS assay. Relative % of IFN-γ+ cells among CD8+ T cells was calculated as follows: % of IFN-γ+ cells with peptide–% of IFN-γ+ cells without peptide. (TIF) [file ppat.1009177.s002.tif]

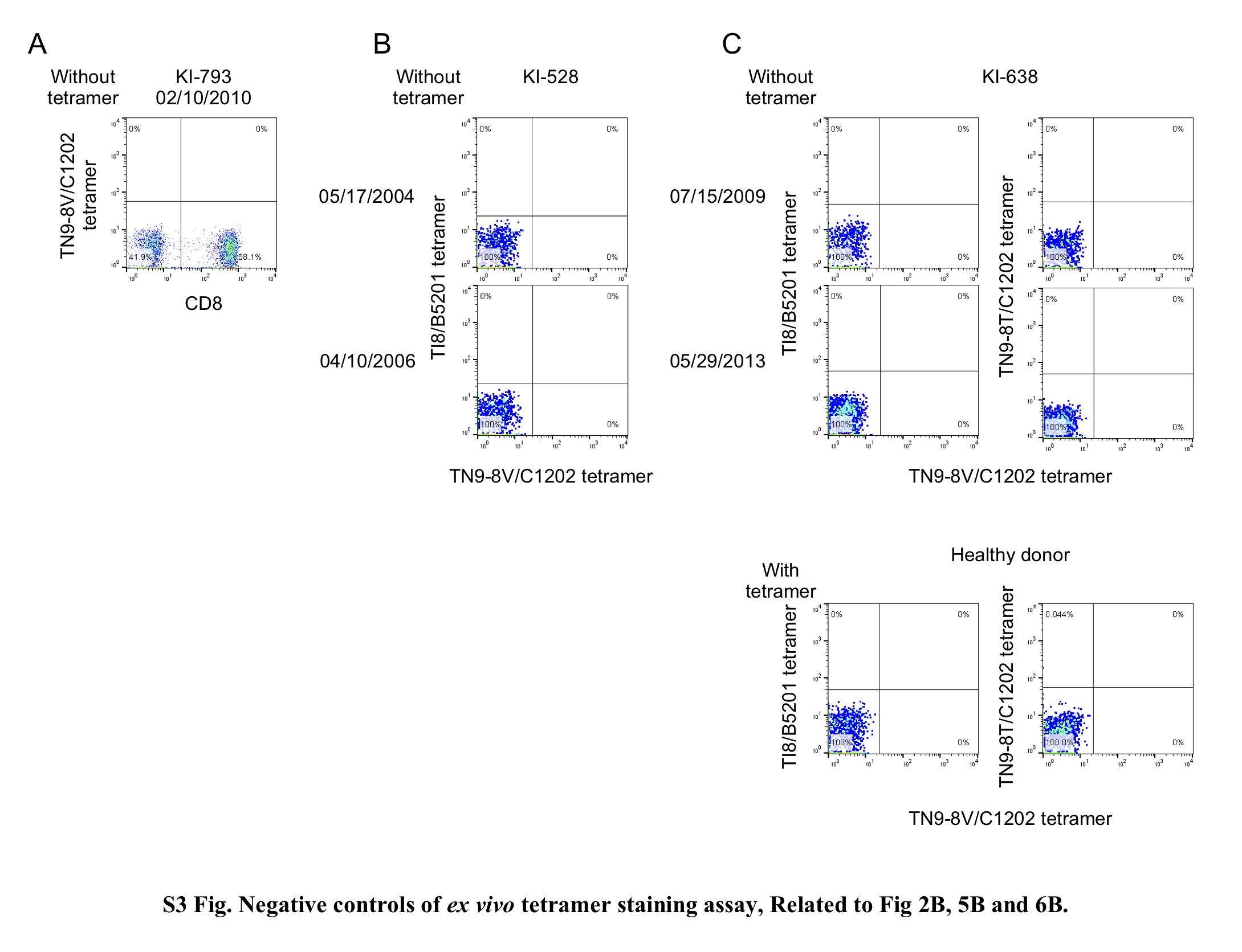

Supplement: S3 Fig — A. Staining of KI-793 PBMCs without tetramer, related to Fig 2B. B. Staining of KI-528 PBMCs without tetramers, related to Fig 5B. C. Staining of KI-638 PBMCs without tetramers (upper) and staining of PBMCs derived from a healthy donor with tetramers (bottom), related to Fig 6B. (TIF) [file ppat.1009177.s003.tif]

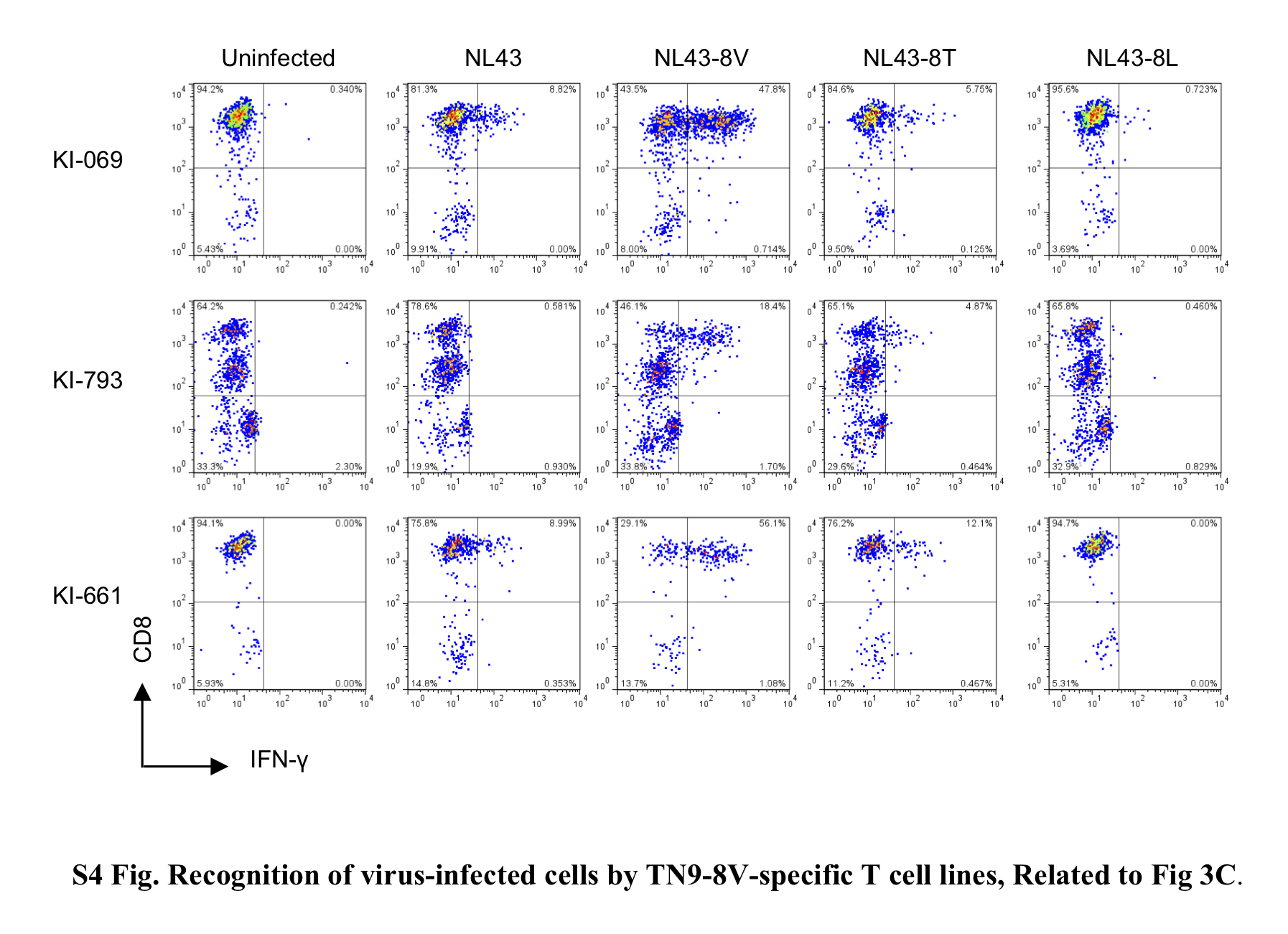

Supplement: S4 Fig — Responses by TN9-8V-specific CTL lines were established from 3 HLA-B*52:01+C*12:02+ individuals with chronic HIV-1. The ability of these T cells to recognize 721.221-CD4-C1202 cells infected with NL43, or NL43-RT135X mutant viruses was analyzed by ICS assay. Frequency of IFN-γ+ cells among CD8+ cells was indicated. (TIF) [file ppat.1009177.s004.tif]

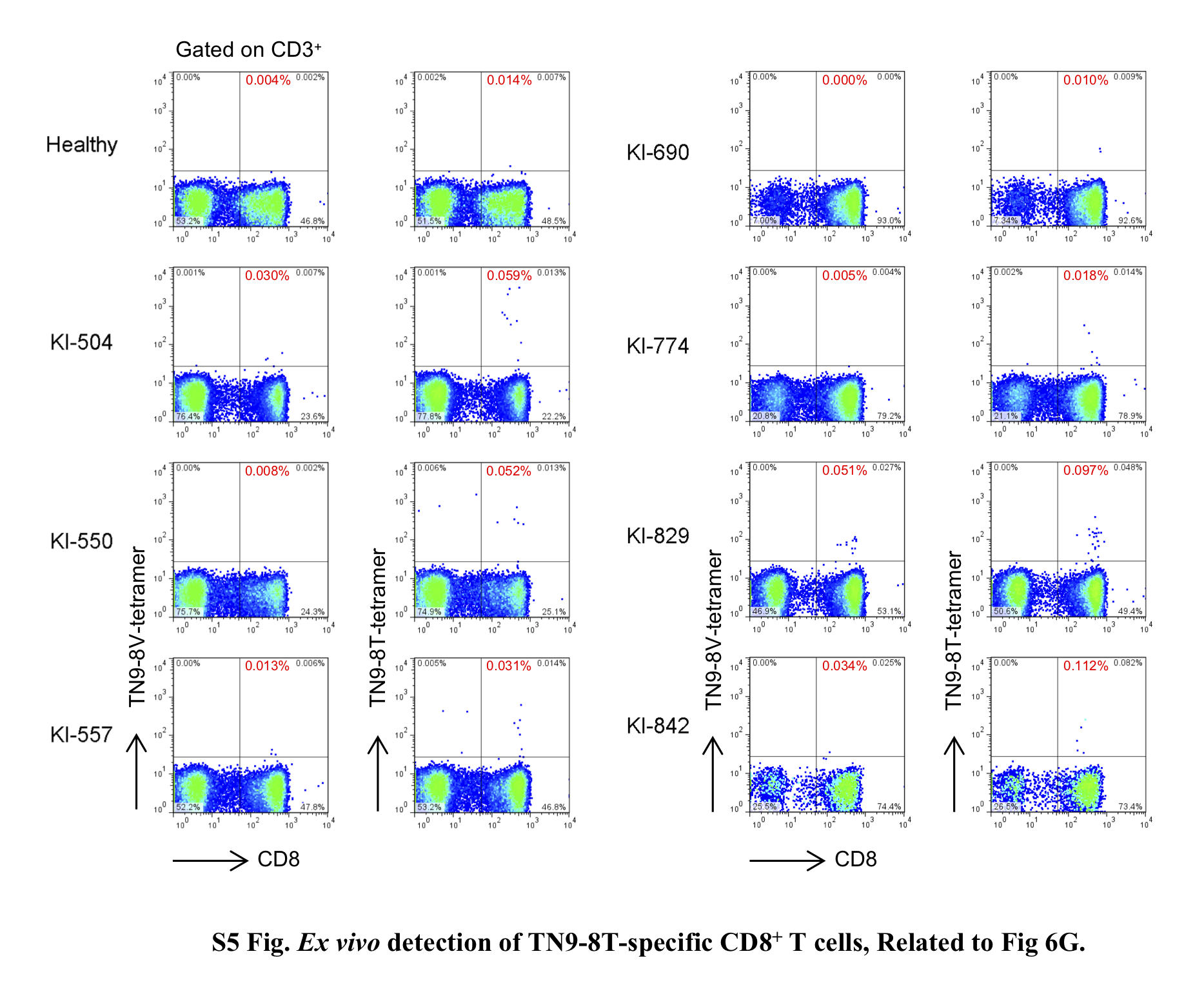

Supplement: S5 Fig — PBMCs from seven HLA-B*52:01+ C*12:02+ individuals harboring HIV-1 RT135T virus were stained with TN9-8V/C1202 tetramer or TN9-8T one at concentration of 100 nM. Representative cases corresponding to Fig 6G were shown. Frequency of tetramer+ cells among CD3+CD8+ T cells is indicated in red. (TIF) [file ppat.1009177.s005.tif]
