# Supplementary material for: T-cell responses to sequentially emerging viral escape mutants shape long-term HIV-1 population dynamics
Source: PLoS Pathog. 2020 Dec 28;16(12):e1009177. doi: 10.1371/journal.ppat.1009177 (PMC7833229; doi:10.1371/journal.ppat.1009177)
Supplement: S1 Table — (DOCX) [file ppat.1009177.s006.docx]

**S1 Table. Codon usages of amino acids at RT135 observed in Japanese hemophiliacs and non-hemophiliacs with chronic HIV-1, Related to S1 Fig.**

| Amino acid |  | Hemophiliacs | | Non-hemophiliacs^a^ | |
| --- | --- | --- | --- | --- | --- |
| at RT135 | Codon | Number | Frequency (%) | Number | Frequency (%) |
| I | ata | 46 | 100 | 20 | 100 |
| V | gta | 17 | 100 | 6 | 100 |
| T | aca  acc  act | 24 | 100 | 32  1  1 | 94  3  3 |
| L | tta | 1 | 100 | 15 | 83 |
|  | cta |  |  | 3 | 17 |

^a^ Non-hemophiliacs were diagnosed with HIV-1 infection before 1997.
