# Supplementary material for: T-cell responses to sequentially emerging viral escape mutants shape long-term HIV-1 population dynamics
Source: PLoS Pathog. 2020 Dec 28;16(12):e1009177. doi: 10.1371/journal.ppat.1009177 (PMC7833229; doi:10.1371/journal.ppat.1009177)
Supplement: S2 Table — (DOCX) [file ppat.1009177.s007.docx]

|  | Amino acid |  | Period | | | |  |  |
| --- | --- | --- | --- | --- | --- | --- | --- | --- |
| HLA carriage | at RT135 |  | -1997 | 1998-2003 | 2004-2009 | 2010-2015 | Total | *p* value |
| All | T | Number | 34 | 96 | 138 | 144 | 412 | 0.0005 |
|  |  | Freq.(%) | 41 | 33 | 26 | 25 |  |  |
|  | Non T | Number | 49 | 191 | 396 | 430 | 1066 |  |
|  |  | Freq.(%) | 59 | 67 | 74 | 75 |  |  |
| B51(-)B52(-) | T | Number | 20 | 59 | 83 | 85 | 247 | 0.0010 |
|  |  | Freq.(%) | 38 | 31 | 24 | 22 |  |  |
|  | Non T | Number | 32 | 131 | 257 | 297 | 717 |  |
|  |  | Freq.(%) | 62 | 69 | 76 | 78 |  |  |
| B51(+)B52(-) | T | Number | 8 | 24 | 30 | 27 | 89 |  |
|  |  | Freq.(%) | 73 | 50 | 35 | 40 |  |  |
|  | Non T | Number | 3 | 24 | 56 | 40 | 123 |  |
|  |  | Freq.(%) | 27 | 50 | 65 | 60 |  |  |
| B51(-)B52(+) | T | Number | 6 | 13 | 25 | 32 | 76 |  |
|  |  | Freq.(%) | 30 | 27 | 23 | 26 |  |  |
|  | Non T | Number | 14 | 36 | 83 | 93 | 226 |  |
|  |  | Freq.(%) | 70 | 73 | 77 | 74 |  |  |
| All | L | Number | 18 | 62 | 192 | 254 | 526 | <0.0001 |
|  |  | Freq.(%) | 22 | 22 | 36 | 44 |  |  |
|  | Non L | Number | 65 | 225 | 342 | 320 | 952 |  |
|  |  | Freq.(%) | 78 | 78 | 64 | 56 |  |  |
| B51(-)B52(-) | L | Number | 11 | 40 | 110 | 176 | 337 | <0.0001 |
|  |  | Freq.(%) | 21 | 21 | 32 | 46 |  |  |
|  | Non L | Number | 41 | 150 | 230 | 206 | 627 |  |
|  |  | Freq.(%) | 79 | 79 | 68 | 54 |  |  |
| B51(+)B52(-) | L | Number | 2 | 13 | 41 | 24 | 80 |  |
|  |  | Freq.(%) | 18 | 27 | 48 | 36 |  |  |
|  | Non L | Number | 9 | 35 | 45 | 43 | 132 |  |
|  |  | Freq.(%) | 82 | 73 | 52 | 64 |  |  |
| B51(-)B52(+) | L | Number | 5 | 9 | 41 | 54 | 109 |  |
|  |  | Freq.(%) | 25 | 18 | 38 | 43 |  |  |
|  | Non L | Number | 15 | 40 | 67 | 71 | 193 |  |
|  |  | Freq.(%) | 75 | 82 | 62 | 57 |  |  |

**S2 Table. Statistical analysis using Cochran-Mantel-Haenszel test on the association between the frequency of 7 amino acids and the four periods, Related to Fig 4.**

**S2 Table. (Continued…)**

|  | Amino acid |  | Period | | | |  |  |
| --- | --- | --- | --- | --- | --- | --- | --- | --- |
| HLA carriage | at RT135 |  | -1997 | 1998-2003 | 2004-2009 | 2010-2015 | Total | *p* value |
| All | V | Number | 6 | 53 | 69 | 71 | 199 | 0.3809 |
|  |  | Freq.(%) | 7 | 18 | 13 | 12 |  |  |
|  | non V | Number | 77 | 234 | 465 | 503 | 1279 |  |
|  | V | Freq.(%) | 93 | 82 | 87 | 88 |  |  |
| B51(-)B52(-) | V | Number | 4 | 31 | 45 | 50 | 130 | 0.3421 |
|  |  | Freq.(%) | 8 | 16 | 13 | 13 |  |  |
|  | non V | Number | 48 | 159 | 295 | 332 | 834 |  |
|  |  | Freq.(%) | 92 | 84 | 87 | 87 |  |  |
| B51(+)B52(-) | V | Number | 0 | 8 | 8 | 6 | 22 |  |
|  |  | Freq.(%) | 0 | 17 | 9 | 9 |  |  |
|  | non V | Number | 11 | 40 | 78 | 61 | 190 |  |
|  | V | Freq.(%) | 100 | 83 | 91 | 91 |  |  |
| B51(-)B52(+) | V | Number | 2 | 14 | 16 | 15 | 47 |  |
|  |  | Freq.(%) | 10 | 29 | 15 | 12 |  |  |
|  | non V | Number | 18 | 35 | 92 | 110 | 255 |  |
|  | V | Freq.(%) | 90 | 71 | 85 | 88 |  |  |
| All | I | Number | 20 | 68 | 111 | 73 | 272 | <0.0001 |
|  |  | Freq.(%) | 24 | 24 | 21 | 13 |  |  |
|  | non I | Number | 63 | 219 | 423 | 501 | 1206 |  |
|  | I | Freq.(%) | 76 | 76 | 79 | 87 |  |  |
| B51(-)B52(-) | I | Number | 17 | 58 | 92 | 57 | 224 | <0.0001 |
|  |  | Freq.(%) | 33 | 31 | 27 | 15 |  |  |
|  | non I | Number | 35 | 132 | 248 | 325 | 740 |  |
|  | I | Freq.(%) | 67 | 69 | 73 | 85 |  |  |
| B51(+)B52(-) | I | Number | 0 | 0 | 2 | 2 | 4 |  |
|  |  | Freq.(%) | 0 | 0 | 2 | 3 |  |  |
|  | non I | Number | 11 | 48 | 84 | 65 | 208 |  |
|  | I | Freq.(%) | 100 | 100 | 98 | 97 |  |  |
| B51(-)B52(+) | I | Number | 3 | 10 | 17 | 14 | 44 |  |
|  |  | Freq.(%) | 15 | 20 | 16 | 11 |  |  |
|  | non I | Number | 17 | 39 | 91 | 111 | 258 |  |
|  | I | Freq.(%) | 85 | 80 | 84 | 89 |  |  |
