# Supplementary material for: T-cell responses to sequentially emerging viral escape mutants shape long-term HIV-1 population dynamics
Source: PLoS Pathog. 2020 Dec 28;16(12):e1009177. doi: 10.1371/journal.ppat.1009177 (PMC7833229; doi:10.1371/journal.ppat.1009177)
Supplement: Attachment — Submitted filename: Reply to reviewers comments.docx [file ppat.1009177.s008.docx]

**Reply to comments from Reviewer#1**

1. In the manuscript entitled “T-cell responses to sequentially emerging viral escape mutants shape long-term HIV-1 population dynamics”, Akahoshi et al. demonstrated HIV-1 coevolved with multiple virus-specific T cells in a host population specific manner, indicating dynamic HIV sequence changes at population level. Specifically, HIV-1 reverse transcriptase codon 135 (RT135) mutant RT135V was selected by HLA-B*52:01-restricted T cells and created a new epitope restricted by HLA-C*12:02. The authors identified novel HLA-C*12:02-restricted T cell epitopes at RT135 and investigated the role of these epitope-specific T cells in selecting RT135 mutants among HLA- B*52:01+ C*12:02+ population. Future, the authors investigated dynamic changes of RT135 mutations over 20 years, in the context of HIV long-term coevolution with multiple virus-specific T cells. The virus-specific T cells were validated at the functional level using cytokine staining and tetramer staining, not just at the sequence level alone. Overall, this is a very interesting and elegant study tracking viral-host interactions and the immune selection pressure at a population level at the scale of decades.

**Reply: Thank you for your very positive evaluation on our paper.**

**Minor issues**

2. The authors may more explicitly point out hemophiliac (figure 1a) and non-hemophiliac (figure 1d) individuals in the figure and figure legend (figure 1d)

**Reply: Based on this comment, we revised Fig.1a and Fig 1b (Fig1d in a previous version) and the legends of these figures.**

3. Given that tetramer staining may be tricky due to the low frequency of positive cells, do the authors have negative control plots for figure 1b, figure 2b, figure 5b, and figure 6b?

**Reply: We showed negative control plots (staining data of PBMCs from the same individuals with anti-CD8 mAb but not with tetramers) for Figs 2B, 5B and 6B in supplement Fig 3. We also showed staining data of PBMC from HIV-1 seronegative individual with the tetramers as negative control for Fig6B in supplemental Fig 3C. The data of PBMC from HIV-1 seronegative individuals with the tetramers for Fig 2B and Fig5B had already been shown in supplemental Fig 5.**

4. Figure 7 legend should include some brief description of the co-evolution of HIV-1 with virus-specific T cells.

**Reply: We moved a brief description explaining Figure 7 from Discussion section to the legend.**

5. The authors may discuss the impact of dynamic population-level changes of HIV-1 sequences and how observed HIV RT135 mutations affect T cell-mediate virus control.

**Reply: We had already discussed this issue in an original manuscript, but we rewrote this part in the fourth paragraph (lines 387-406) to clarify this.**

**Reply to comments from Reviewer#2**

1. In the study by Akahoshi et al, the authors present a study consisting of population-level virology and immunology. They demonstrate HLA associations and viral evolution patterns of HIV-1 epitope escape variants. This study compares viral and immune features between 95 hemophiliacs and 1,478 non-hemophiliac HIV-1-infected men. The inclusion of hemophiliacs is very clever, as it represents a "control" population presumably initially infected with WT virus. This study is well-controlled, the experiments are properly performed, and it presents some very interesting findings that are of relevance to the field. Despite this, there are several issues, mostly associated with narrative choices and style, that detract from the study.

**Reply: Thank you for your very positive evaluation on our paper. We revised the manuscript based on your comments described specifically below.**

**Minor issues**

2. The manuscript in general is very dense, wordy, and difficult to follow, with diffuse goals. It could benefit from a very clear goal. Much of the excess verbiage should be trimmed to focus only on the key messages that are integral to the main point of the paper.

**Reply: We revised and shortened the introduction, result, and discussion sections based on this comment.**

3. Similarly, the abstract should be clear and concise, and convey a central message

**Reply: We revised the abstract and author summary based on this comment.**

4. Much of the introduction could be omitted, moved to the discussion, or condensed

**Reply: Based on this comment, we revised and shorted the introduction section.**

5. In some cases, figure panels and comparisons can be combined or rearranged. For example, Figures 1A and D make more sense to be discussed back to back. Figure 1B and C seem superfluous and could realistically be moved to supplementary.

**Reply: Based on this comment, we deleted Figure 1B because this data is shown in Figure 2C. Figure 1C was moved to supplementary (Supplemental Figure 1). We also deleted a part explaining the data shown in Fig1B and moved a part explaining the data shown Fig1C to next part in Result section (line 152-156).**

**Reply to comments from Reviewer#3**

1. Akahoshi et al present a manuscript with the title: T-cell responses to sequentially emerging viral escape mutants shape long-term HIV-1 population dynamics.They show that HIV-1 strains harboring escape mutations selected by virus-specific cytotoxic T lymphocytes accumulate at the population level. In this context they investigated population-level changes for a single epitope at HIV-1 Reverse Transcriptase codon 135 (RT135), which is under pressure by T cells restricted by two different HLA-B alleles that are frequent in Japan. They found that RT135V selection by HLA-B*52:01-restricted T-cells resulted in the emergence of a new epitope restricted by HLA-C*12:02. Their results suggest multiple virus-specific T cells as dynamic drivers of population-level HIV-1 evolution. While the study is of interested for the specific understanding of HIV evolution under immune pressure its design is extremely narrow. The authors study the evolution and immune pressure for only one T cell epitope (RT135). The HLA alleles they focus on are especially frequent in Japan but not, for example, in Canada. Thus, the study reports a very specific finding, which is probably not of interest for a wide range of scientists in the field of infectious diseases, but rather for HIV researchers only. It would therefore be more appropriate for a specialized HIV journal. Along these lines, the generalized statement at the end of the abstract: “Together, our observations indicate that host population-specific T-cell responses to sequentially emerging viral escape mutants can shape long-term HIV-1 population dynamics, thus demonstrating HIV-1 coevolution with multiple virus-specific T cells.“ sounds interesting and meaningful, but has already been shown by several previous publications. Thus, the current study does not provide findings that are novel and outstanding enough to justify publication in a journal that is reflecting the full breadth of infectious diseases, like PLoS Pathogens.

**Reply: Thank you for your critique for this study. We showed the case in Japanese individuals with HIV-1 in this study, but we believe that this is very important example to understand coevolution of HIV-1 with T cells. Similar cases at other HIV-1 positions may exist in individuals with HIV-1 in other countries or other cohorts and in other infectious diseases. We therefore believe that this paper provides a strong impact on studies of various infectious diseases.**
